# Differential gene expression analysis of ‘Chili’ (*Pyrus bretschneideri*) fruit pericarp with two types of bagging treatments

**DOI:** 10.1038/hortres.2017.5

**Published:** 2017-03-08

**Authors:** Yuling Wang, Xinfu Zhang, Ran Wang, Yingxin Bai, Chenglian Liu, Yongbing Yuan, Yingjie Yang, Shaolan Yang

**Affiliations:** 1 Department of Horticulture, Qingdao Agricultural University, Qingdao 266109, China; 2 Qingdao Key Laboratory of Genetic Improvement and Breeding in Horticultural Plants, Qingdao 266109, China

## Abstract

Preharvest bagging is a simple, grower-friendly and safe physical protection technique commonly applied to many fruits, and the application of different fruit bags can have various effects. To explore the molecular mechanisms underlying the fruit quality effects of different bagging treatments, digital gene expression (DGE) profiling of bagged and unbagged ‘Chili’ (*Pyrus bretschneideri* Rehd.) pear pericarp during development was performed. Relative to unbagged fruit, a total of 3022 and 769 differentially expressed genes (DEGs) were detected in the polyethylene (PE)-bagged and non-woven fabric-bagged fruit, respectively. DEGs annotated as photosynthesis-antenna proteins and photosynthesis metabolism pathway were upregulated in non-woven fabric-bagged fruit but downregulated in the PE-bagged fruit. Non-woven fabric bagging inhibited lignin synthesis in ‘Chili’ pear pericarp by downregulating DEGs involved in phenylpropanoid biosynthesis; consequently, the fruit lenticels in non-woven fabric-bagged fruit were smaller than those in the other treatments. The results indicate that the non-woven fabric bagging method has a positive effect on the appearance of ‘Chili’ pear fruit but neither of the two bagging treatments is conducive to the accumulation of soluble sugar.

## INTRODUCTION

‘Chili’ (*Pyrus bretschneideri* Rehd.) pear fruit is native to China and has an obovate shape, yellow-green skin and a recessed calyx. It is a successful cultivar of Asian pear with a high sugar content and juicy flesh, but the fruit of the ‘Chili’ pear has large fruit lenticels and a rough pericarp, which limits its popularity. Regarding the mechanisms of ‘Chili’ pear fruit lenticel formation, Liu *et al.*^1^ suggested that the stoma of pear fruit is destroyed during fruit development and that the parenchyma in the cavity of the stoma resumes dividing and forms the phellogen. The phellogen then produces cork cells that gradually appear on the skin of the fruit, and a fruit lenticel forms, indicating that formation of the fruit lenticel is related to the production of cork cells. Many studies have shown that lignin and other phenolic substances are structural components of cork cells,^2,3^ but there are few reports regarding the relationship between lignin and the fruit lenticel.

Preharvest bagging improves the skin color, avoids biological and abiotic stress, and changes the microenvironment of fruit development, which has multiple effects on fruit quality.^4–6^ Faoro *et al.*^7^ reported that the use of a small transparent paraffin paper bag or large brown craft paper bag for ‘Housui’ pears resulted in a better appearance, as the fruit were more uniform in size and had smooth, shiny skin with small lenticels. Similarly, Lin *et al.*^8^ suggested that paper-bagged ‘Cuiguan’ and ‘Hosui’ pears were brighter and more attractive, with fewer visible russet dots than non-bagged fruit. Although much research has been performed on the effect of bagging on the pear fruit quality of various cultivars, the type of bag recommended for one fruit may not work well for another fruit.^9^ The ideal type of bag for ‘Chili’ pear fruit is unknown.

Polyethylene (PE) bags have been widely used in orchards because of their lower cost and good light transmission. Amarante *et al.*^6^ reported that ‘Doyenne du Comice’ pears bagged with micro-perforated PE bags ~30 days after full bloom showed greener and lighter skin color than non-bagged fruit. However, considering environmental issues, the development of biodegradable bags is required. Some researchers also have shown beneficial results using paper bags, but the use of such bags in areas of heavy rainfall may not be feasible.^4^ In recent years, non-woven fabric bags have been used for fruit because they are waterproof, have good air permeability and light transmission, and are made from a recyclable and environmentally friendly material.^10^ The ‘Red Globe’ and ‘Muscat Hamburg’ grape cultivars show good color and brightness when bagged with non-woven fabric bags before harvest, and the levels of soluble solid, anthocyanin and vitamin C in the berries are higher than when paper bags are used.^10^ In peaches, white non-woven polypropylene bagging treatment improves color development.^11^ Due to the advantages of non-woven fabric bags, we explored the mechanism of how these bags affect pears to contribute to developing a better bag material to obtain a favorable external quality of the pears.

The complex process of fruit quality development involves various genes and metabolic pathways. Whole-genome sequencing of pears has been reported;^12,13^ thus, RNA-Seq analysis of pears to study fruit quality and regulation mechanisms are feasible. To explore the molecular mechanisms underlying the effects of different bagging treatment on pear fruit quality and to uncover which specific metabolic processes influence fruit quality, we used RNA-Seq to explore differentially expressed genes (DEGs) in the ‘Chili’ pear with two types of bagging treatments: PE and non-woven fabric bags.

## MATERIALS AND METHODS

### Plant materials

Pear fruits from 15-year-old trees (*Pyrus bretschneideri* Rehd. cv. Chili) at a farm near Laiyang (36°58′N, 120°43′E, Shandong, China) were bagged with PE or non-woven fabric bags on day 60 after anthesis. The irrigation and fertilization conditions were appropriate and identical throughout the orchard. We designed three treatments: (i) no bags (control); (ii) green PE bags (manufactured by Laiyang Xintai Fruit Bag Company, China), with dimensions of 160×160 mm^2^, a single thickness of 6.875 μm, and 88.76% transparency, which was measured by a Lux Meter (ZDS-10, Shanghai, China); and (iii) white non-woven polypropylene fabric bags (manufactured by Qingdao Wonong Modern Agricultural Limited Company, China), with dimensions of 180×180 mm^2^, a single thickness of 210 μm and 66.47% transparency. Thirty pear fruits were equally divided into three experimental groups: bagged into PE bags or non-woven fabric bags or left unbagged on day 60, 75, 90, 105, 120, 135, 150, 165 and 180 (harvest day) after anthesis, respectively. The pericarp of unbagged, PE-bagged and non-woven fabric-bagged fruit on 150 and 180 days after anthesis was cut into ~1cm^2^ pieces, mixed, treated with liquid nitrogen and stored at −70 °C for further assays and sequencing. The samples collected at 150 days after anthesis treated with no bags, PE bags or non-woven fabric bags were designated E1, E3 and E5, respectively. Similarly, the corresponding samples collected at 180 days after anthesis were designated E2, E4 and E6, respectively.

### Measurement of lignin content

The lignin content was determined according to a previously published method and calculated based on absorbance at 280 nm with an ultraviolet spectrophotometer (Beijing, PERSEE, China).^14^ A solution of NaOH was used as a control. The lignin content was expressed as 10^3^A280 per kg dry weight (DW) for three replicates.

### Measurement of soluble sugar content

The anthrone colorimetric method was used to determine the soluble sugar content according to Li *et al.*^15^ Soluble sugar was extracted from the fruit peel as follows: Fresh samples (100–300 mg) were boiled in 5–10 ml distilled water for 30 min two times. The extracting solution was filtered into a 25 ml volumetric flask, and 1 ml was transferred into a test tube, followed by the addition of 1.5 ml of distilled water. Detection was performed according to the above method for three replicates.

### RNA-Seq protocol

Total RNA was extracted from the samples using an RNA extraction kit (Omega, Georgia, USA) and treated with DNase I (Fermentas, Vilnius, Lithuania) according to the instructions of the manufacturer. High quality total RNA (5 μg) was purified with Oligo (dT) magnetic beads and then broken into short fragments. Using these mRNA fragments as templates, first-strand and second-strand cDNA was synthesized. Next, a single ‘A’ base was added to the 3′ end of the repaired cDNA fragments, and Illumina paired-end solexa adapters were subsequently ligated to these cDNA fragments. The size of templates was selected by agarose gel electrophoresis. PCR was performed to enrich the purified cDNA template. Finally, the six libraries generated from the samples described above (E1–E6) were sequenced using an Illumina HiSeq 2500 system at the Biomarker Technologies Corporation (Beijing, China). In this study, the RNA-Seq project for ‘Chili’ pear was initiated (NCBI BioProject Accession: SRP063324, http://www.ncbi.nlm.nih.gov/bioproject/PRJNA294723). In the following analysis, the RNA-Seq results of the samples on 150 and 180 days after anthesis were analyzed.

### Bioinformatic analysis and quantitative real-time PCR validation

After removing reads for which the ratio of the unknown base ‘N’ was >10% and other low-quality reads (that is, a quality score of <10), clean reads were filtered from the raw reads. Q30, the proportion of nucleotides with quality values >30, and the GC content, the proportion of guanine and cytosine nucleotides among total nucleotides, were calculated. The clean reads were then mapped to the pear reference genome (https://www.rosaceae.org/species/pyrus/pyrus_communis/genome_v1.0) using TopHat version 1.4.1.^16^ The mismatch value was 2, and the other parameters were set to the default. The sequence alignment files generated by TopHat were provided as input to the software Cufflinks,^17^ which was used to estimate the gene expression level. The number of clean reads for each gene was calculated and then normalized as reads per kilobase of exon model per million mapped reads (RPKM), using the method described by Mortazavi *et al.*^18^

Because there were no replicates in this study, the DEGs were defined using EBSeq software,^19^ and there were no >50% DEGs in the data set. A false discovery rate (FDR) of<0.01 and a fold change of ⩾2 were selected as cutoffs to identify significantly different gene expression. GO enrichment analysis of DEGs was performed using topGO.^20^ GO terms with adjusted *P*-values of <0.05 were defined as significantly enriched GO terms. KEGG (Kyoto Encyclopedia of Genes and Genomes) pathway enrichment analysis was performed using KOBAS based on the adjusted *P*-value of <0.05.^21^

The samples of ‘Chili’ pear fruit at 60, 120, 150 and 180 days after anthesis were selected for quantitative real-time PCR (q-PCR) assays. Total RNA was extracted using RNA plant Plus Reagent (TIANGEN, Beijing, China) according to the manufacturer’s instructions. DNA contamination was removed using DNase I (Fermentas). The first cDNA strand was reverse transcribed using a Revert Aid First Strand cDNA Synthesis kit (Fermentas) according to the manufacturer’s instructions.

Q-PCR was performed using a Light Cycler 480 instrument (Roche, Basel, Switzerland). The reaction volumes of 20 μl included 2 μl of cDNA, 0.4 μl of each primer and 10 μl of 2×SYBR Green PCR Master Mix (Roche). The primer set used for q-PCR analysis was designed by Primer 3 (http://primer3.ut.ee/). Pear *actin* was used as an internal control to normalize small differences in template amounts. Primer sequences of the target genes and *actin* for q-PCR are shown in Supplementary Table S1. The q-PCR protocol included annealing at 94 °C for 5 min, followed by 40 cycles of 94 °C for 15 s and 60 °C for 1 min. A negative control without template for each primer pair was included in each run. Relative expression levels were calculated using the 2^-ΔΔCt^ method and normalized to the *actin* gene.^22^ There were three replicates for each gene.

### Statistical analyses

Standard errors were calculated using Origin software (Northampton, MA, USA). The least significant differences shown in the figures were calculated by DPS version 7.05 (*α*=0.05). Pearson’s correlation analysis of gene expression between RNA-Seq and q-PCR was performed using SPSS version 17.0 (IBM, Armonk, New York, USA).

## RESULTS

### Characterization of the ‘Chili’ pear fruit with different bagging treatments

**Figure 1:**
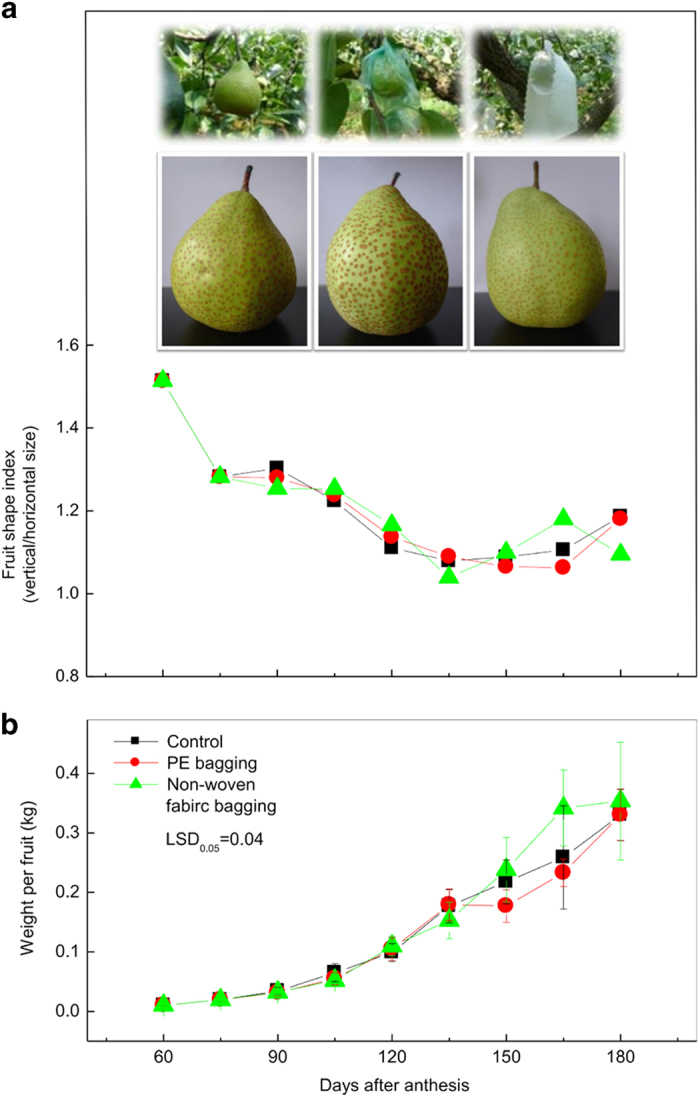
Growth and development of ‘Chili’ pear fruit with different bagging treatments. (**a**) The fruit shape index (vertical/horizontal diameter) of unbagged, PE-bagged (bagged 60 days after anthesis) and non-woven fabric-bagged (bagged 60 days after anthesis) ‘Chili’ pear fruit during development. The pictures show unbagged (left), PE-bagged (middle) and non-woven fabric-bagged (right) ‘Chili’ pear fruit 120 days (up) and 180 days (down) after anthesis. (**b**) The weight per fruit of unbagged, PE-bagged and non-woven fabric-bagged ‘Chili’ pear fruit during development.

The fruit shape index (vertical/horizontal diameter) and weight per fruit were measured at 60, 75, 90, 105, 120, 135, 150, 165 and 180 days after anthesis. The fruit shape index decreased during fruit growth and development, particularly at 60–75 and 105–135days after anthesis, which suggested that the fruit expanded rapidly at these two stages. No significant difference was observed between bagged and unbagged fruit (Figure 1a). The fruit weight increased slowly at prophase and then rapidly from 90 days after anthesis until the fruit ripened. Consequently, the growth curves of ‘Chili’ pear had an ‘S’ shape (Figure 1b). Moreover, the weight of non-woven fabric-bagged fruit showed a tendency of more rapid growth than PE-bagged fruit and unbagged fruit between 150 and 180 d, but the fruit weight among the different treatments was similar at harvest. The above results indicate that different bagging treatments have no influence on ‘Chili’ pear fruit size or weight. Amarante *et al.*^6^ also reported that preharvest PE bagging had no effect on ‘Doyenne du Comice’ pear fruit size, weight or maturity, which is consistent with our results. However, the pears bagged with non-woven fabric bags did show a cleaner, smoother and brighter appearance than PE-bagged fruit or unbagged fruit at harvest (Figure 1a).

### Illumina sequencing evaluation analysis

After filtering the adaptor sequences and removing low-quality tags, we obtained 61.33 M reads. The total number of bases was 3.13 G, and the Q30 percentage (sequences with sequencing error rate lower than 0.1%) was over 87%. The average GC content was 47.69% (Supplementary Table S2). To ensure the reliability of the libraries, we performed quality control and obtained 10,147,862 (E1), 10,538,550 (E2), 9,575,078 (E3), 10,604,295 (E4), 10,372,779 (E5) and 10,095,380 (E6) clean reads after trimming (Supplementary Table S2). These data showed that the Illumina sequencing was of high quality. Among the total cleaned reads, 8,557,466 (E1), 8,901,940 (E2), 8,099,640 (E3), 8,940,804 (E4), 8,605,947 (E5) and 8,385,706 (E6) were mapped to the *Pyrus communis* genome with mapping ratios of 84.33% (E1), 84.47% (E2), 84.59% (E3), 84.31% (E4), 82.97% (E5), and 83.06% (E6) (Supplementary Table S3). All the data indicated that the sequencing quality was sufficiently high for further analysis.

### Comparison and analysis of DEGs

**Figure 2:**
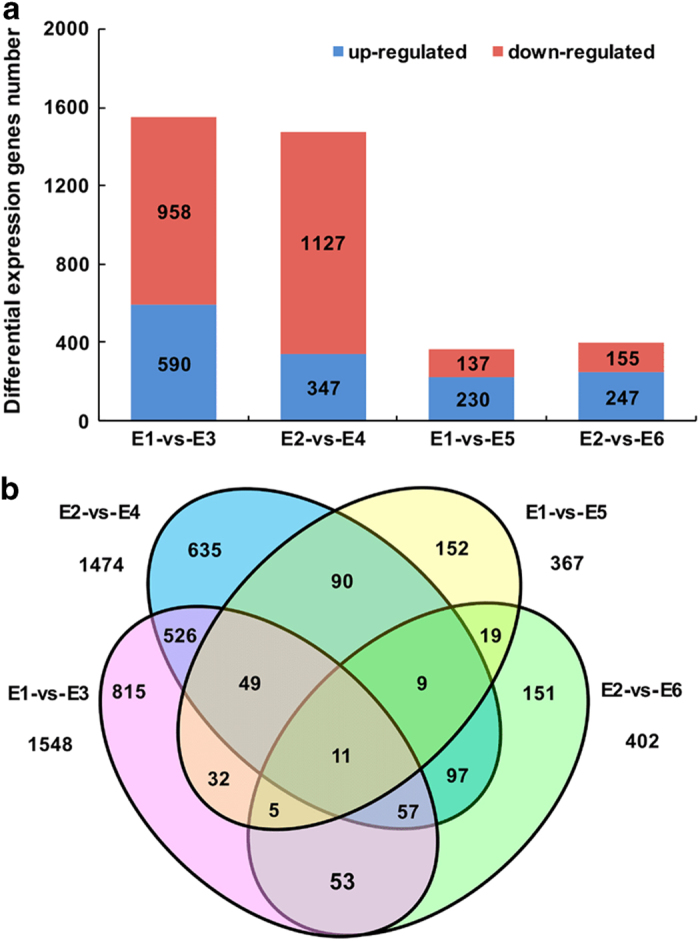
Summary of DEGs. (**a**) The DEGs of bagged fruit relative to unbagged fruit. (**b**) DEGs shown in Venn diagram. E1-versus-E3 represents the PE-bagged ‘Chili’ fruit versus unbagged fruit 150 days after anthesis, E1-versus-E5 represents the non-woven fabric-bagged ‘Chili’ fruit versus unbagged fruit 150 days after anthesis, E2-versus-E4 represents the PE-bagged ‘Chili’ fruit versus unbagged fruit 180 days after anthesis, and E2-versus-E6 represents the non-woven fabric-bagged ‘Chili’ fruit versus unbagged fruit 180 days after anthesis.

A total of 1,548 (958 upregulated, 590 downregulated) and 1,474 (1,127 upregulated, 347 downregulated) DEGs were detected in the PE-bagged fruit versus unbagged fruit at 150 days and 180 days after anthesis, respectively. For the non-woven fabric-bagged fruit versus unbagged fruit, 367 (137 upregulated, 230 downregulated) and 402 (155 upregulated, 247 downregulated) DEGs were detected at 150 days and 180 days after anthesis, respectively (Figure 2a). The majority of the DEGs were upregulated in the PE-bagged fruit and downregulated in the non-woven fabric-bagged fruit. Venn diagram results indicated that 643 DEGs overlapped between 150 days and 180 days in the PE-bagged fruit, whereas only 44 DEGs overlapped between 150 days and 180 days in the non-woven fabric-bagged fruit, and 11 DEGs overlapped between PE-bagged fruit and non-woven fabric-bagged fruit (Figure 2b).

### GO and KEGG enrichment analysis of DEGs

**Figure 3:**
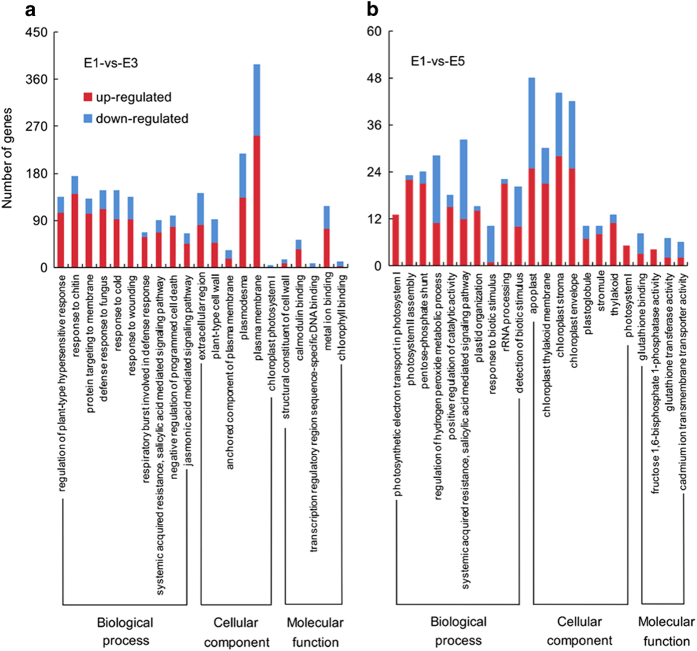
GO enrichment of DEGs between bagged fruit and unbagged fruit 150 days after anthesis. E1-versus-E3 represents the PE-bagged ‘Chili’ fruit versus unbagged fruit 150 days after anthesis, and E1-versus-E5 represents the non-woven fabric-bagged ‘Chili’ fruit versus unbagged fruit 150 days after anthesis.

The top 10 enriched GO terms corresponding to DEGs detected in the two bagging treatments are shown in Figure 3 (GO terms less than 10 are all listed). For the DEGs between PE-bagged and unbagged fruit at 150 days after anthesis, the GO terms related to stress response and plant hormone signal transduction were significantly enriched, and most of the DEGs were upregulated in PE-bagged fruit. Regarding molecular function, GO terms related to cell composition were significantly enriched, and most DEGs were upregulated. In addition, chloroplast photosystem I was also enriched, whereas the DEGs were all downregulated in PE-bagged fruit. Interestingly, most of the DEGs in chlorophyll binding (in the category of cellular component) were also downregulated (Figure 3a). GO term enrichment of the DEGs in PE-bagged fruit at 180 days after anthesis was similar to these results (Supplementary Figure S1).

Comparing the DEGs between non-woven fabric-bagged and unbagged fruit at 150 days after anthesis, the biological process GO terms related to photosynthesis, stress response and plant hormone signal transduction were significantly enriched; for molecular function, the significantly enriched GO terms were almost all related to photosynthesis (Figure 3b). This analysis showed that most of the DEGs related to photosynthesis were upregulated in the non-woven fabric-bagged fruit, in contrast to the PE-bagged fruit. The GO term enrichment of the DEGs in non-woven fabric-bagged fruit at 180 days after anthesis showed a similar pattern (Supplementary Figure S1).

**Figure 4:**
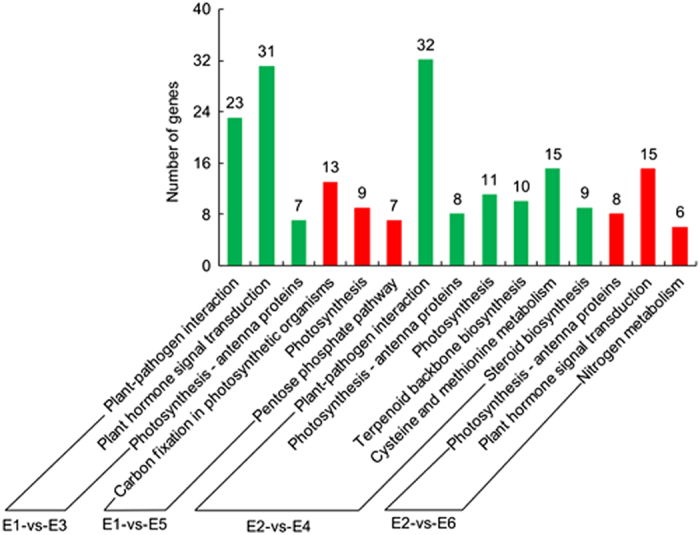
KEGG enrichment of DEGs between bagged fruit and unbagged fruit. E1-versus-E3 represents the PE-bagged ‘Chili’ fruit versus unbagged fruit 150 days after anthesis, E1-versus-E5 represents the non-woven fabric-bagged ‘Chili’ fruit versus unbagged fruit 150 days after anthesis, E2-versus-E4 represents the PE-bagged ‘Chili’ fruit versus unbagged fruit 180 days after anthesis, and E2-versus-E6 represents the non-woven fabric-bagged ‘Chili’ fruit versus unbagged fruit 180 days after anthesis.

To compare the specific metabolic pathways in which DEGs participated between bagged and unbagged fruit, KEGG pathway enrichment analysis was performed. Three pathways were significantly enriched for the DEGs between PE-bagged and unbagged fruit at 150 days after anthesis: plant-pathogen interaction (corrected *P*-value=2.27×10^-4^, 23 genes), plant hormone signal transduction (3.68×10^-4^, 31) and photosynthesis-antenna proteins (1.28×10^-2^, 7; Figure 4). Comparing the DEGs between PE-bagged and unbagged fruit at 180 days after anthesis, the pathways of plant-pathogen interaction (6.76×10^-10^, 32), photosynthesis-antenna proteins (1.71×10^-3^, 8), photosynthesis (1.02×10^-2^, 11), terpenoid backbone biosynthesis (1.80×10^-2^, 10), cysteine and methionine metabolism (2.26×10^-2^, 15) and steroid biosynthesis (2.41×10^-2^, 19) were highly enriched.

Comparing the DEGs between non-woven fabric-bagged and unbagged fruit at 150 days after anthesis, the most highly enriched pathways were carbon fixation in photosynthetic organisms (1.88×10^-6^, 13), photosynthesis (4.28×10^-5^, 9) and pentose phosphate pathway (7.67×10^-3^, 7). Comparing the DEGs between non-woven fabric-bagged and unbagged fruit at 180 days after anthesis, there were three significantly enriched pathways: photosynthesis-antenna proteins (2.92×10^-7^, 8), plant hormone signal transduction (1.98×10^-3^, 15) and nitrogen metabolism (3.18×10^-3^, 6).

The GO and KEGG enrichment analysis suggested that the primary differences between bagged and unbagged fruit were the fruit color, taste and the photosynthetic factors. All DEGs involved in the above metabolic pathways can be found in Supplementary Table S4. The DEGs in the photosynthesis pathway annotated as *Psb27*, *PsbR*, *PsbS*, *PsaF*, *PsaG*, *PsaN*, *PsaK* and *gamma* (GDR accession no: PCP036514, PCP027351, PCP010956, PCP033216, PCP007354, PCP025193, PCP044408 and PCP040275), genes encoding photosynthesis-antenna proteins annotated as *Lhcb4*, *Lhcb6*, *Lhca2* and *Lhca4* (GDR accession no: PCP017020, PCP000701, PCP018057 and PCP005206), genes involved in starch and sucrose metabolism annotated as *SPS* and *Inv* (GDR accession no: PCP030726, PCP030531), and three genes involved in phenylpropanoid biosynthesis annotated as *Pp4CL*, *PpCAD* and *PpPOD* (GDR accession no: PCP018170, PCP015510 and PCP010039) were selected for further analysis. To confirm the results of the RNA-Seq analysis, the relative expression levels of DEGs of interest were validated by q-PCR. The q-PCR results of DEGs that are not discussed in the following text are shown in Supplementary Figure S2. In addition, correlation analysis of DEGs expression pattern between RNA-Seq and q-PCR, which indicated that the correlation between RNA-Seq and q-PCR was significant, is shown in Supplementary Table S5.

### Genes related to photosynthesis-antenna proteins and photosynthesis

**Figure 5:**
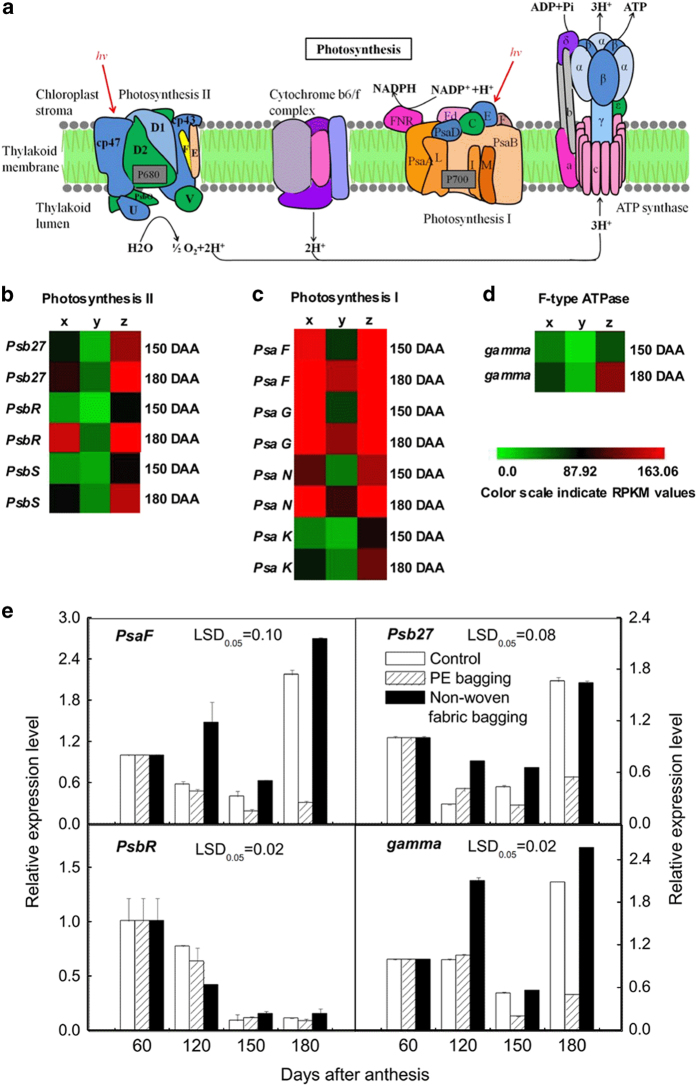
The photosynthesis of ‘Chili’ pear with different bagging treatments. (**a**) The pathway of photosynthesis-antenna proteins (based on the KEGG pathway, http://www.kegg.jp/kegg-bin/show_pathway?map00195). (**b**) Photosystem II gene expression patterns are indicated by RPKM values. (**c**) Photosystem I gene expression patterns are indicated by RPKM values. (**d**) F-type ATPase gene expression patterns are indicated by RPKM values. (**e**) Gene expression level is inferred by q-PCR. Error bars on each column indicate SEs from three replicates. The expression pattern of each DEG is shown by 3 grids: the left one represents the RPKM value of the unbagged fruit (x), the middle one represents the RPKM value of the PE-bagged fruit (y) and the right one represents the RPKM value of the non-woven fabric-bagged fruit (z). The grids with different colors from green to red show the absolute expression magnitude with the RPKM values 0–163.06. DAA, days after anthesis.

Photosynthesis in green plants is the process of using light energy to synthesize organic compounds from carbon dioxide and water, a series of reactions that consists of photosystem II (PSII), cytb6 complex, photosystem I (PSI) and ATP synthase (ATPase; Figure 5a).

**Figure 6:**
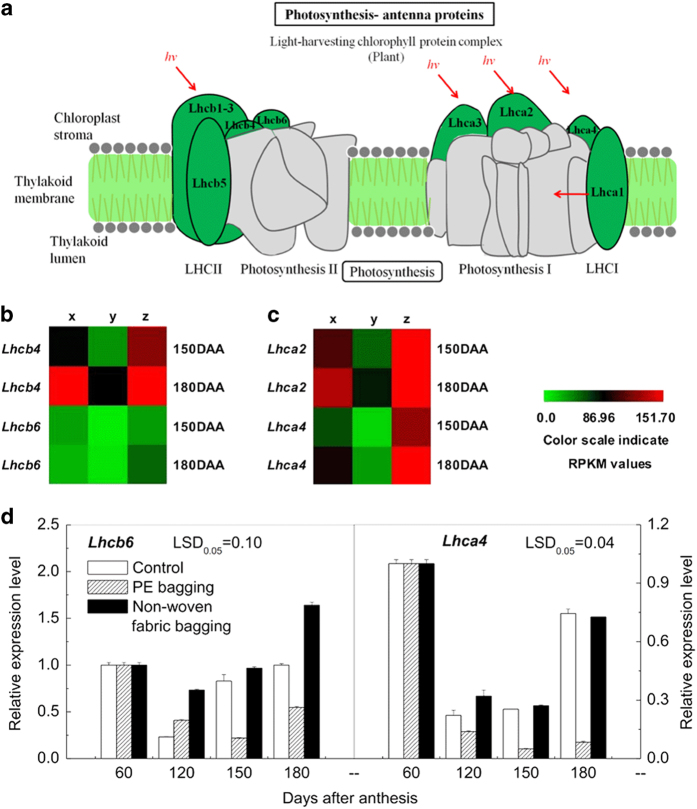
The photosynthesis-antenna proteins of ‘Chili’ pear with different bagging treatments. (**a**) The pathway of photosynthesis-antenna proteins (based on KEGG pathway, http://www.kegg.jp/dbget-bin/www_bget?map00196). (**b**) Expression pattern of genes involved in LHCII are indicated by RPKM values. (**c**) Expression pattern of genes involved in LHCI are indicated by RPKM values. (**d**) Gene expression level inferred by q-PCR. Error bars on each column indicate SEs from three replicates. The expression pattern of each DEG is shown by 3 grids: the left one represents the RPKM value of the unbagged fruit (x), the middle one represents the RPKM value of the PE bagging fruit (y), and the right one represents the RPKM value of the non-woven fabric-bagged fruit (z). The grids with different colors from green to red show the absolute expression magnitude with the RPKM values 0–151.70. DAA, days after anthesis.

Photosystem II is the site where oxygen is generated for plant growth and development.^23^ Three DEGs (*Psb27*, *PsbR* and *PsbS*) encoding reaction center proteins of PSII and two DEGs (*Lhcb4* and *Lhcb6*) encoding chloroplast pigment-binding proteins CP29 and CP24 of the light-harvesting pigment complex II (LHCII) were upregulated in the non-woven fabric-bagged fruit but downregulated in the PE-bagged fruit (Figures 5b and 6b), consistent with the results of q-PCR (Figures 5e and 6d). The above results suggest that the capacity for light harvest and oxygen release in non-woven fabric-bagged fruit may be larger than that of PE-bagged fruit.

Photosystem I is an important part of the photosynthetic machinery that catalyzes transmembrane electron transfer via plastocyanin/ferredoxinoxido-reductase activity and produces NADPH for CO_2_ assimilation.^24^ In the present study, the genes of *PsaF*, *PsaG*, *PsaK*, *PsaN*, *Lhca2* and *Lhca4* were all downregulated in PE-bagged fruit but upregulated in non-woven fabric-bagged fruit (Figures 5c and 6c); the q-PCR results closely matched the RNA-Seq results (Figures 5e and 6d).

H^+^-ATP synthase is a multi-subunit protein formed by one γ, three α, and three β subunits.^25^ The γ subunit, which is the central rotor, constitutes the rotating ‘shaft’ that mediates energy exchange between the proton and ATP. In this study, *gamma*, the gene that encodes the γ subunit, was downregulated in the PE-bagged fruit, whereas it was upregulated in the non-woven fabric-bagged fruit (Figure 5d), consistent with the q-PCR results (Figure 5e).

### Genes related to starch and sucrose metabolism

**Figure 7:**
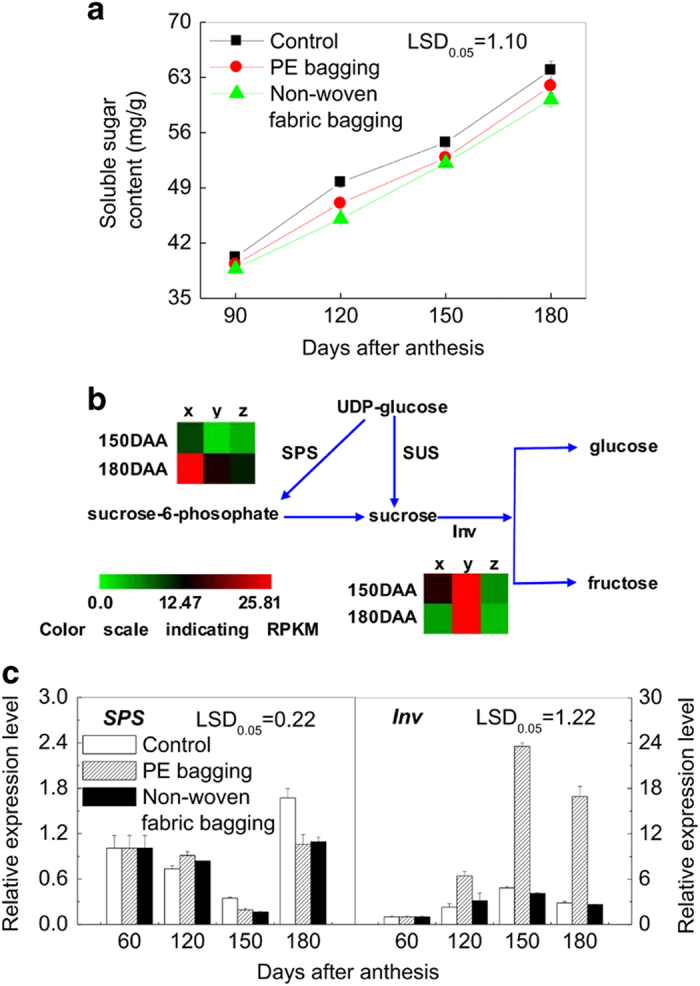
Starch and sucrose metabolism of ‘Chili’ pear with different bagging treatments. (**a**) The soluble sugar content of ‘Chili’ fruit pericarp during development. Error bars on each symbol indicate SEs from three replicates. (**b**) The pathway of starch and sucrose metabolism (http://www.kegg.jp/dbget-bin/www_bget?map00500). (**c**) Gene expression level inferred by q-PCR. Error bars on each column indicate SEs from three replicates. The expression pattern of each DEG is shown by 3 grids: the left one represents the RPKM value of the unbagged fruit (x), the middle one represents the RPKM value of the PE-bagged fruit (y), and the right one represents the RPKM value of the non-woven fabric-bagged fruit (z). The grids with different colors from green to red show the absolute expression magnitude with the RPKM values 0–25.81. DAA means days after anthesis.

In this study, the content of total soluble sugar in the PE-bagged fruit and non-woven fabric-bagged fruit pericarp both increased with fruit growth and development (Figure 7a), although they were all lower relative to unbagged fruit, consistent with a previous report.^26^ The expression of the SPS gene (Figure 7c) was downregulated by PE and non-woven fabric bagging treatments, and there was no significant difference between these two groups of bagged fruit, which is consistent with the RNA-Seq results (Figure 7b). The gene expression pattern of Inv in the PE-bagged fruit was significantly higher than that of non-woven fabric-bagged fruit and unbagged fruit but did not show a direct correlation with soluble sugar content (Figure 7c).

### Genes related to lignin synthesis

**Figure 8:**
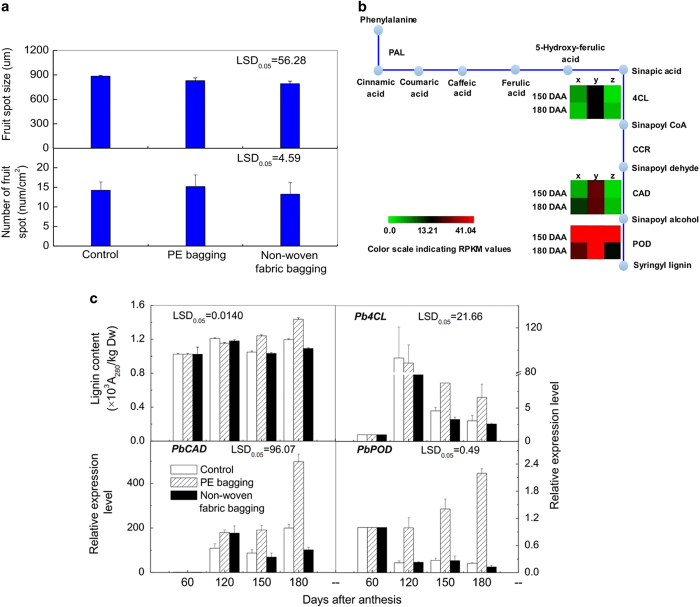
The lignin synthesis metabolism of ‘Chili’ pear with different bagging treatments. (**a**) The fruit lenticel size and number of unbagged, PE-bagged and non-woven fabric-bagged fruit on harvest day. (**b**) The phenylpropanoid biosynthesis pathway (http://www.kegg.jp/dbget-bin/www_bget?map00940). Lignin synthesis gene expression patterns are indicated by RPKM values. (**c**) Gene expression level inferred by q-PCR. Error bars on each column indicate s.e. from three replicates. The expression pattern of each DEG is shown by three grids: the left one represents the RPKM value of the unbagged fruit (x), the middle one represents the RPKM value of the PE-bagged fruit (y), and the right one represents the RPKM value of the non-woven fabric-bagged fruit (z). The grids with different colors from green to red show the absolute expression magnitude with the RPKM values 0–41.04. DAA means days after anthesis.

The extent of lignification is an important factor in the quality of many fruits, such as Whangkeumbae pear^14^ and loquat,^27^ because it decreases the fresh market value. A previous study showed that the fruit lenticels are lignified cells above the fruit skin.^1^ In this study, preharvest bagging treatments had no detectable influence on the number of fruit lenticels. However, the non-woven fabric bagging technique decreased the fruit lenticel diameter significantly, whereas the fruit lenticel size of PE-bagged fruit was similar to that of the unbagged fruit (Figure 8a). In addition, PE-bagged fruit had higher lignin content than that of unbagged fruit during the late period of fruit development, although the non-woven fabric-bagged fruit showed the opposite pattern (Figure 8c).

The biosynthesis of lignin is a complex process in plants that is reportedly associated with the enzyme activities of phenylalanine ammonia-lyase (PAL), 4-coumaroyl-CoA synthetase (4CL), cinnamyl alcohol dehydrogenase (CAD) and peroxidase (POD).^28^ In the pathway of phenylpropanoid metabolism, the genes of *Pb4CL*, *PbCAD* and *PbPOD*, which encode the key enzymes 4CL, CAD and POD, were all upregulated in PE-bagged fruit but downregulated in non-woven fabric-bagged fruit (Figure 8b), consistent with the RNA-Seq analysis (Figure 8c).

## DISCUSSION

In this study, we present the first reported gene expression profile of the pericarp of differently bagged ‘Chili’ pears during development. Specifically, we focused on the effects of different bagging treatments on fruit quality by examining the specific metabolic processes and DEGs involved. The results show that fruit bagging affects photosynthesis and the syntheses of soluble sugar and lignin in the ‘Chili’ pear pericarp. The genes annotated in these metabolic processes showed different regulation patterns with different bag types, and the results of our study suggest that non-woven fabric bags have a positive effect on fruit quality.

Fruit lenticels are a significant factor that influences fruit appearance. In this study, the lenticels of non-woven fabric-bagged fruit were smaller than the control, and the lenticels of the PE-bagged fruit were the largest among all the groups. A previous study suggested that the formation of fruit lenticels is related to lignin.^1^ The lignin content in PE-bagged fruit was higher than that of non-woven fabric-bagged fruit, which is consistent with our observation of fruit lenticel size. The enzymes 4CL, CAD and POD are important in lignin synthesis. 4CL is the branch point enzyme that channels general phenylpropanoid metabolism into specific lignin biosynthesis branches, which catalyzes the activation of hydroxycinnamic acids into their corresponding coenzyme A esters.^29^ Some studies have found that the lignin content decreases after *4CL* downregulation,^30,31^ and this may be one of the reasons that the PE-bagged fruit contains more lignin. CAD catalyzes the last step of monolignol biosynthesis by converting the cinnamoyl-CoA esters into monolignols.^32^ It has also been reported that *OsCAD2* and *OsCAD7* affect monolignol biosynthesis.^33,34^ Thus, the upregulation of *PbCAD* could cause the lignin content to increase in PE-bagged fruit. POD is involved in the oxidative polymerization of monolignols to form lignin, the last major step in lignin synthesis.^35^ An antisense construct of the POD gene *Shpx6a* transferred into poplar significantly reduced the lignin content of the leaves by 10–20%.^36^ Thus, the lower lignin content in non-woven fabric-bagged fruit may be related to the downregulation of *PbPOD*. These results indicate that different fruit bags affect the fruit lenticel size of ‘Chili’ pear fruit by regulating the expression pattern of these genes. In addition, Chen *et al.*^37^ reported that higher levels of lignin are correlated with increased anionic peroxidase activity in light-treated mungbean tissues. We inferred that the high lignin content in PE-bagged fruit pericarp could be related to the strong light transmittance of the PE bag. However, the lignin content in unbagged fruit pericarp was lower than that of PE-bagged fruit, which may have been caused by the wavelength of the impinging light or other factors. Further study is needed to determine the specific mechanism responsible for these effects.

In a previous study, it was suggested that fruit photosynthesis can provide a significant proportion of the carbon requirement of reproduction and may positively contribute to the entire plant carbon budget, and green peels showed remarkable photosynthetic activity.^38^ Enrichment analysis showed that GO terms and KEGG pathways related to photosynthesis overlapped between the two groups of fruit with different bagging treatments. Most of the DEGs that were associated with the GO terms ‘chloroplast thylakoid membrane’, ‘photosystem I’ and ‘photosystem II’ were downregulated in PE-bagged fruit but upregulated in non-woven fabric-bagged fruit; DEGs enriched in the KEGG pathways ‘photosynthesis-antenna proteins’ and ‘photosynthesis’ showed a similar pattern. These results suggest that photosynthesis may be more active in the non-woven fabric-bagged fruit than the PE-bagged fruit. We infer that the PE bagging treatment decreases the light-harvesting ability of ‘Chili’ fruit and then inhibits photosynthesis. In addition, we assume that the depression of photosynthesis in PE-bagged fruit is related to the low air permeability of the PE bags. The non-woven fabric-bagged fruit showed higher photosynthetic capacity than PE-bagged fruit, which may be due to the superior air permeability and the CO_2_ provided by this bag. In addition, the wavelength of light through the fruit bag may be different, which would also influence the photosynthetic rate. Understanding the mechanism underlying these differences requires further exploration.

Neither of the two bagging treatments was conducive to the accumulation of soluble sugar. Feng *et al.*^39^ reported that the total sugar content of loquat fruit decreases as the transparency of paper bags declines. Therefore, we hypothesize that the lower sugar content in bagged fruit may be due to a decrease in irradiance. Moriguchi *et al.*^40^ inferred that SPS may play a critical role in sucrose accumulation in Asian pears. Pattanayak^41^ proposed that a change in irradiance plays a pivotal role in regulating the activation of SPS in potato and also showed that the maximum SPS activity is coincident with a high level of irradiance. Therefore, we infer that the bagging treatment reduces irradiance, decreasing the gene expression level of *SPS* and inhibiting the synthesis of sucrose, as reported in bagged ‘Conference’ and ‘Placer’ pears.^42,43^

## CONCLUSIONS

This study is the first to use gene expression profile to reveal the effect of different bagging treatments on pear fruit quality. The results suggest that non-woven fabric bags are better suited than PE bags for ‘Chili’ pear fruit. In addition, the results clarify the relationship between lignin and the fruit lenticel; non-woven fabric bags reduce the diameter of fruit lenticels by suppressing lignin synthesis-related gene expression. Moreover, this study suggests that the non-woven fabric bags have a positive effect on fruit photosynthesis by upregulating the relative expression level of genes. Here we show that preharvest non-woven fabric bagging treatment significantly improves the appearance quality of ‘Chili’ pears.
