# Supplementary material for: Differential gene expression analysis of ‘Chili’ (Pyrus bretschneideri) fruit pericarp with two types of bagging treatments
Source: Hortic Res. 2017 Mar 8;4:17005–. doi: 10.1038/hortres.2017.5 (PMC5341540; doi:10.1038/hortres.2017.5)
Supplement: Supplementary Table S1 [file hortres20175-s1.doc]

Molecular function

**A**

Number of genes

E2-vs-E4 represents the PE-bagged ‘Chili’ fruit versus unbagged fruit on 180 days after anthesis, E2-vs-E6 represents the non-woven fabric-bagged ‘Chili’ fruit versus unbagged fruit on 180 days after anthesis, GO: 0016706 represents the GO term ‘oxidoreductase activity, acting on paired donors, with incorporation or reduction of molecular oxygen, 2-oxoglutarate as one donor, and incorporation of one atom each of oxygen into both donors’.

**Figure S1** GO enrichment of DEGs between bagged fruit and unbagged fruit on 180 days after anthesis.

Molecular function

Cellular component

Biological process

Cellular component

Biological process

**B**

E2-vs-E6

E2-vs-E4
