# Supplementary material for: Differential gene expression analysis of ‘Chili’ (Pyrus bretschneideri) fruit pericarp with two types of bagging treatments
Source: Hortic Res. 2017 Mar 8;4:17005–. doi: 10.1038/hortres.2017.5 (PMC5341540; doi:10.1038/hortres.2017.5)
Supplement: Supplementary Table S2 [file hortres20175-s2.doc]

**Figure S2** Q-PCR validation of DEGs

**Genes related to photosynthesis and photosynthesis antenna proteins**

**Genes related to starch and sucrose metabolism**

**Genes related to carotenoid biosynthesis****Genes related to carbon fixation in photosynthetic organisms**

**Genes related to plant hormane signal transduction**
