# Supplementary material for: Differential gene expression analysis of ‘Chili’ (Pyrus bretschneideri) fruit pericarp with two types of bagging treatments
Source: Hortic Res. 2017 Mar 8;4:17005–. doi: 10.1038/hortres.2017.5 (PMC5341540; doi:10.1038/hortres.2017.5)
Supplement: Supplementary Table S3 [file hortres20175-s3.doc]

**Table S1** Primers used for q-PCR analysis.

| **Gene name** | **Primer name** | **Primer sequence** |
| --- | --- | --- |
| *actin*  *PsbR*  *Psb27*  *PsaF*  *gamma*  *Lhca4*  *Lhcb6*  *Pp4CL*  *PpPOD*  *PpCAD* | actin-F  actin-F  PsbR-F  PsbR-R  Psb27-F  Psb27-R  PsaF-F  PsaF-R  gamma-F  gamma-R  Lhca4-F  Lhca4-R  Lhcb6-F  Lhcb6-R  Pp4CL-F  Pp4CL-R  PpPOD-F  PpPOD-R  PpCAD-F  PpCAD-R | 5′- CCCAGAAGTGCTCTTCCAAC -3′  5′- TTGATCTTCATGCTGCTTGG -3′  5′- ATTCAAGGTGCAAGCCAGTG -3′  5′- CCCCAGCATAGACATCACCA -3′  5′- CACAATCCCACAACCTCGAC -3′  5′- GGCCACCCATGAGTTTGATG -3′  5′- GACTCACCCCATGCAAAGAC -3′  5′- ATCGGACCCACAAAGCAAAC -3′  5′- GTTTGAGCAGGATCCGGTTC -3′  5′- GTTGGCACCCGAAACAATCT -3  5′- TTCTTCGTCCACCCTCTTCG -3′  5′- CTCGAGAGTGGGTTCGAAGT -3  5′- AAGAAGTCAGGCCTTGCTCT -3′  5′- CGCCTCTCTGTACCACTTGA -3′  5′- GCAGCCAAATCATGAAAGGT -3′  5′- GCCACTTGGAACCCTTTGTA -3′  5′- TGGACTCAAGGCCAACTTCT -3′  5′- ATTCTGCGGGCATATCTCAC -3′  5′- TTTGGTTGAGAGAGTTGCCCAC -3′  5′- ATTCGACACCCAAGCTCTTCG -3′ |
