# Supplementary material for: Differential gene expression analysis of ‘Chili’ (Pyrus bretschneideri) fruit pericarp with two types of bagging treatments
Source: Hortic Res. 2017 Mar 8;4:17005–. doi: 10.1038/hortres.2017.5 (PMC5341540; doi:10.1038/hortres.2017.5)
Supplement: Supplementary Table S4 [file hortres20175-s4.doc]

**Table S2** Throughput and quality of RNA-Seq of ‘Chili’ pear.

| **ID** | **Raw reads** | **Clean reads** | **Total nucleotides (bp)** | **GC percentage** | **Q30 percentage** |
| --- | --- | --- | --- | --- | --- |
| E1 | 10,213,817 | 10,147,862 | 517,468,762 | 47.98% | 88.30% |
| E2 | 10,622,703 | 10,538,550 | 537,393,177 | 47.75% | 88.34% |
| E3 | 9,676,261 | 9,575,078 | 488,258,497 | 47.66% | 89.02% |
| E4 | 10,685,873 | 10,604,295 | 540,743,817 | 47.58% | 88.39% |
| E5 | 10,469,157 | 10,372,779 | 528,938,070 | 47.55% | 87.59% |
| E6 | 10,180,796 | 10,095,380 | 514,792,864 | 47.59% | 87.25% |

E1: unbagged fruit on 150 days after anthesis, E2: unbagged fruit on 180 days after anthesis, E3: PE-bagged fruit on 150 days after anthesis, E4: PE-bagged fruit on 180 days after anthesis, E5: non-woven fabric-bagged fruit on 150 days after anthesis, E6: non-woven fabric-bagged fruit on 180 days after anthesis.
