# Supplementary material for: Differential gene expression analysis of ‘Chili’ (Pyrus bretschneideri) fruit pericarp with two types of bagging treatments
Source: Hortic Res. 2017 Mar 8;4:17005–. doi: 10.1038/hortres.2017.5 (PMC5341540; doi:10.1038/hortres.2017.5)
Supplement: Supplementary Table S5 [file hortres20175-s5.doc]

**Table S3** Mapped results of the six samples with the reference pear genomics.

| **ID** | **Clean reads** | **Mapped reads**  **(Mapped ratio)** | **Unique mapped reads(Mapped ratio)** | **Multiple mapped reads(Mapped ratio)** |
| --- | --- | --- | --- | --- |
| E1  E2  E3  E4  E5  E6 | 10,147,862  10,538,550  9,575,078  10,604,295  10,372,779  10,095,380 | 8,557,466(84.33%)  8,901,940(84.47%)  8,099,640(84.59%)  8,940,804(84.31%)  8,605,947(82.97%)  8,385,706(83.06%) | 7,468,722(87.28%)  7,895,199(88.69%)  7,222,738(89.17%)  7,900,867(88.37%)  7,650,499(88.90%)  7,467,456(89.05%) | 1,088,744(12.72%)  1,006,741(11.31%)  876,902(10.83%)  1,039,937(11.63%)  955,448(11.10%)  918,250(10.95%) |

E1: unbagged fruit on 150 days after anthesis, E2: unbagged fruit on 180 days after anthesis, E3: PE-bagged fruit on 150 days after anthesis, E4: PE-bagged fruit on 180 days after anthesis, E5: non-woven fabric-bagged fruit on 150 days after anthesis, E6: non-woven fabric-bagged fruit on 180 days after anthesis.
