# Supplementary material for: Differential gene expression analysis of ‘Chili’ (Pyrus bretschneideri) fruit pericarp with two types of bagging treatments
Source: Hortic Res. 2017 Mar 8;4:17005–. doi: 10.1038/hortres.2017.5 (PMC5341540; doi:10.1038/hortres.2017.5)
Supplement: Supplementary Figure S1 [file hortres20175-s6.doc]

**Table S4** Candidate DEGs involved in ‘Chili’ fruit quality related metabolism.

| **Pathway** | **Treatment** | | **Gene** | **Protein/Enzyme(EC No.)** | **KO id** | **NO.**  **Up** | **NO.**  **Down** |
| --- | --- | --- | --- | --- | --- | --- | --- |
| **DEGs related to Energy Metabolism** | | | | | | | |
| **Photosynthesis** | | Control vs  PE bagging | PsaF  PsaG  PsaL  PsaN  PsbP  PsbR  Psb27  PetC  PetH  gamma | /  /  /  /  /  /  Photosystem II lipoprotein Psb27  Cytochrome b6-f complex iron-sulfur subunit (1.10.9.1)  Ferredoxin--NADP reductase (1.18.1.2)  H(+)-transporting two-sector ATPase(3.6.3.14) | 02694  02695  02699  02701  02717  03541  08902  02636  02641  02115 | 0  0  0  0  0  0  0  0  0  0 | 1  2  1  2  1  1  1  1  1  2 |
|  |  | Control vs non-woven bagging | PsaK  PsaN  PsbR  PsbS  PetE  PetC  PetH  gamma | /  /  /  /  /  Cytochrome b6/f complex (1.10.99.1)  FNR (1.18.1.2)  H(+)-transporting two-sector ATPase(3.6.3.14) | 02698  02701  03541  03542  02638  02636  02641  02109 | 1  1  1  2  1  1  2  1 | 0  0  0  0  0  0  0  0 |
| **Photosynthesis –antenna proteins** | | Control vs  PE bagging | Lhca2  Lhca3  Lhca4  Lhcb1  Lhcb2  Lhcb4  Lhcb5  Lhcb6 | /  /  /  /  /  /  /  / | 08908  08909  08910  08912  08913  08915  08916  08917 | 0  0  0  0  0  0  0  0 | 1  1  1  3  1  2  1  2 |
|  |  | Control vs non-woven bagging | Lhca3  Lhca4  Lhcb1  Lhcb3  Lhcb6 | /  /  /  /  / | 08909  08910  08912  08914  08917 | 2  2  3  2  1 | 0  0  0  0  0 |
| **Citrate cycle**  **(TCA cycle)** | | Control vs  PE bagging | ACO  ACL | Aconitate hydratase(4.2.1.3)  ATP citrate synthase(2.3.3.8) | 01681  01648 | 1  3 | 0  0 |
|  |  | Control vs non-woven bagging | MDH  FUM | malate dehydrogenase(1.1.1.37)  fumarate hydratase(4.2.1.2) | 00026  01679 | 1  0 | 0  1 |
| **DEGs related to taste development** | | | | | | | |
| **Starch and sucrose metabolism** | | Control vs  PE bagging | PHS  SPS  GLGB  GLGA  GAUT  SCRK  GLGL  PME  CXE  AMY  INV  G6PI | Glycogen phosphorylase(2.4.1.1)  Sucrose-phosphate synthase(2.4.1.14)  1,4-α-glucan branching enzyme(2.4.1.18)  Starch synthase(2.4.1.21)  Polygalacturonate 4-alpha-galacturonosyltransferase(2.4.1.43)  Fructokinase(2.7.1.4)  Glucose-1-phosphate adenylyltransferase(2.7.7.27)  Pectinesterase(3.1.1.11)  Carboxylesterase(3.1.1.12)  α-amylase(3.2.1.1)  β-fructofuranosidase(3.2.1.26)  Glucose-6-phosphate isomerase(5.3.1.9) | 00688  00696  00700  00703  13648  00847  00975  01051  01087  01176  01193  01810 | 0  0  0  1  1  1  1  2  2  1  1  1 | 1  1  1  1  0  0  2  1  0  0  0  0 |
|  |  | Control vs non-woven bagging | SPS  CAUT  INV  CXE  BGL | Sucrose-phosphate synthase (2.4.1.14)  Polygalacturonate4-alpha-galacturonosyltransferase(2.4.1.43)  β-fructofuranosidase(3.2.1.26)  Carboxylesterase (3.1.1.12)  β-glucosidase (3.2.1.21) | 00696  13648  01193  01087  01188 | 0  1  0  1  0 | 1  0  2  0  1 |
| **Fatty acid biosynthesis** | | Control vs  PE bagging | ELH  FabF  FabI | 3-oxoadipate enol –lactonase(3.1.1.24)  /  / | 10782  09458  00208 | 0  0  0 | 1  1  1 |
|  |  | Control vs non-woven bagging | FabI | / | 00208 | 1 | 1 |
| **Phenylalanine metabolism** | | Control vs  PE bagging | POD  PAL  4CL | Peroxidase(1.11.1.7)  Phenylalanine ammonia-lyase(4.3.1.24)  4-coumarateCoA ligase(6.2.1.12) | 00430  10775  01904 | 2  2  1 | 3  1  0 |
|  |  | Control vs non-woven bagging | POD  4CL  PAL | Peroxidase(1.11.1.7)  4-coumarateCoA ligase(6.2.1.12)  Phenylalanine ammonia-lyase(4.3.1.24) | 00430  01904  10775 | 0  0  2 | 2  1  2 |
| **Phenylpropanoid biosynthesis** | | Control vs  PE bagging | POD  PAL  4CL  CAD  CALB  F5H | Peroxidase(1.11.1.7)  Phenylalanine ammonia-lyase(4.3.1.24)  4-coumarateCoA ligase(6.2.1.12)  Cinnamyl-alcohol dehydrogenase(1.1.1.195)  Coniferyl-aldehyde dehydrogenase(1.2.1.68)  Ferulate-5-hydroxylase(1.14.-.-) | 00430 10775 01904  00083  12355  09755 | 2  2  1  1  1  1 | 3  1  0  0  0  0 |
|  |  | Control vs non-woven bagging | POD  4CL  PAL  BGL | Peroxidase(1.11.1.7)  4-coumarateCoA ligase(6.2.1.12)  Phenylalanine ammonia-lyase(4.3.1.24)  β-glucosidase(3.2.1.21) | 00430  01904  10775  01188 | 0  0  2  0 | 2  1  2  1 |
| **DEGs related to color development** | | | | | | | |
| **Carotenoid biosynthesis** | | Control vs  PE bagging | CrtR  NCED  ABA  ABAH | /  9-cis-epoxycarotenoid dioxygenase(1.13.11.51)  Xanthoxin dehydrogenase(1.1.1.288)  (+)-abscisic acid 8'-hydroxylase(1.14.13.93) | 02294  09840  09841  09843 | 2  1  0  1 | 0  2  2  0 |
|  |  | Control vs non-woven bagging | NCED | 9-cis-epoxycarotenoid dioxygenase(1.13.11.51) | 09840 | 2 | 1 |
