# Supplementary material for: Differential gene expression analysis of ‘Chili’ (Pyrus bretschneideri) fruit pericarp with two types of bagging treatments
Source: Hortic Res. 2017 Mar 8;4:17005–. doi: 10.1038/hortres.2017.5 (PMC5341540; doi:10.1038/hortres.2017.5)
Supplement: Supplementary Figure S2 [file hortres20175-s7.doc]

**Table S5** Pearson correlation analysis of gene expression pattern between RNA-Seq and q-PCR.

| Pearson correlation coefficient (r)  Gene name | r value |
| --- | --- |
| *PsaF*  *PsaN* | 0.969  0.814  0.729 |
| *Psb27* |  |
| *PsbR* | 0.584 |
| *gamma*  *Lhcb2* | 0.886  0.932 |
| *Lhcb6*  *Lhca2*  *Lhca3* | 0.951  0.646  0.704 |
| *Lhca4*  *GAUT6*  *Inv*  *SPS* | 0.727  0.710  0.956  0.891 |
| *Pb4CL* | 0.937 |
| *PbCAD* | 0.759 |
| *PbPOD*  *RBSC*  *NCED*  *PP2C6*  *MYC2* | 0.671  0.842  0.991  0.675  0.858 |

r represents pearson correlation coefficient, 0.5< |r| <0.8 represents significant correlation,

0.8< |r| <1 represents high correlation.
